# Event-Oriented State Alignment Network for Weakly Supervised Temporal Language Grounding

**DOI:** 10.3390/e26090730

**Published:** 2024-08-27

**Authors:** Hongzhou Wu, Xiang Zhang, Tao Tang, Canqun Yang, Zhigang Luo

**Affiliations:** School of Computer, National University of Defense Technology, Changsha 410073, China; zhangxiang08@nudt.edu.cn (X.Z.); taotang84@nudt.edu.cn (T.T.); canqun@nudt.edu.cn (C.Y.); zgluo@nudt.edu.cn (Z.L.)

**Keywords:** temporal language grounding, cross-modal, relative entropy, neural networks

## Abstract

Weakly supervised temporal language grounding (TLG) aims to locate events in untrimmed videos based on natural language queries without temporal annotations, necessitating a deep understanding of semantic context across both video and text modalities. Existing methods often focus on simple correlations between query phrases and isolated video segments, neglecting the event-oriented semantic coherence and consistency required for accurate temporal grounding. This can lead to misleading results due to partial frame correlations. To address these limitations, we propose the Event-oriented State Alignment Network (ESAN), which constructs “start–event–end” semantic state sets for both textual and video data. ESAN employs relative entropy for cross-modal alignment through knowledge distillation from pre-trained large models, thereby enhancing semantic coherence within each modality and ensuring consistency across modalities. Our approach leverages vision–language models to extract static frame semantics and large language models to capture dynamic semantic changes, facilitating a more comprehensive understanding of events. Experiments conducted on two benchmark datasets demonstrate that ESAN significantly outperforms existing methods. By reducing false high correlations and improving the overall performance, our method effectively addresses the challenges posed by previous approaches. These advancements highlight the potential of ESAN to improve the precision and reliability of temporal language grounding tasks.

## 1. Introduction

Temporal language grounding (TLG) is a multi-modal task that aims to find events in untrimmed videos by matching a natural language query and outputting the starting and ending times of the video segment. Unlike temporal action localization, which retrieves information based on a finite set of predefined actions such as “run” or “drink”, temporal language grounding (TLG) operates through open-domain natural language sentences. This approach requires models to deeply understand the semantic context in both video and text modalities to achieve accurate grounding. TLG is particularly effective for interpreting mixed and complex events due to its ability to process an infinite variety of sentence queries. For instance, the capability to handle a query like “A close-up of a glass door is shown, along with a person’s hand moving around in the camera and making various gestures” demonstrates TLG’s ability to address such complex scenarios, thereby endowing it with broader applicability. This requires the model to not simply learn specific patterns but to delve deeply into understanding the connection between natural language queries and video content. Some works [1,2,3] have introduced training under fully supervised setting. However, this requires extensive human effort and time for temporal annotations. Therefore, some researchers [4,5] have turned to weakly supervised TLG, where training samples only provide “video–text” pairs without annotated starting and ending times for the matching segments. Previous works [6,7] have conducted cross-modal alignment by treating untrimmed videos as positive bags in multiple-instance learning. Recently, reconstruction-based approaches [8,9,10] have been proposed for weakly supervised TLG, which regards the video segment that reconstructs the query better as more relevant to the query. However, the problem is that the aforementioned methods primarily focus on correlations between query phrases and isolated video segments, neglecting event-oriented semantic coherence and consistency across modalities. This oversight can cause models to be misled by similar irrelevant frames. So our research question is how to solve the spurious high correlations of similar irrelevant frames.

**Figure 1:**
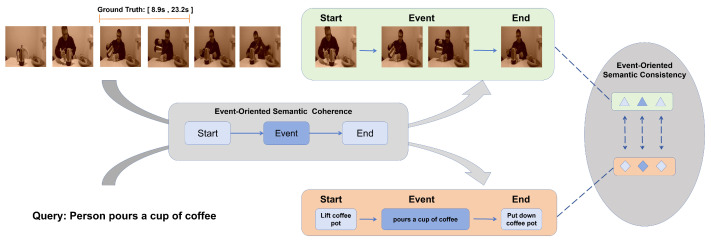
An example of event-oriented semantic coherence and consistency in temporal language grounding. The event described in the query and the corresponding video segment can be transformed into sets of semantic state changes as a “start–event–end” pattern. These semantic sets are intra-modally semantically coherent. Due to them being sets of semantic states in different modalities about the same event, they satisfy cross-modal consistency in semantics.

In fact, when humans understand the events taking place in a video, they grasp how an event causes objects to change from their initial state to their final state [11]. For example, as shown in Figure 1, for the query “person pours a cup of coffee”, the viewer not only understands the action itself but also understands its start state may be “a person picks up a coffee pot” and the end state would be “a person puts down the coffee pot”. Inspired by this insight, we hypothesize that only video segments where the start state, event action, and end state all align with the query should be considered valid matches, while confusing frames only relate to specific words in the query but fail to capture the complete state transitions. That is to say, a model needs to comprehend the sequence of state changes—“start–event–end”—to truly understand the essence of the event described in the query, rather than merely identifying superficial similarities between words and frame elements. This necessitates multi-modal features that ensure intra-modal semantic coherence and cross-modal semantic consistency throughout the state transition process.

To address this issue, we propose the Event-oriented State Alignment Network (ESAN) for improved cross-modal alignment and inference. First, we construct a cross-modal semantic state space. In this space, query events from the textual modality are transformed into a “start–event–end” state set. This state set needs to be semantically coherent, meaning that its start and end states are closely related to the event itself. For the video modality, due to the fact that temporal annotations are not available for hard alignment in weakly supervised TLG, we use the model’s predicted proposals as soft labels. We then project the video features within the proposed start and end times into the cross-modal semantic state space. We believe that the projection of video features for a correct proposal can meet semantic coherence, while incorrect proposals often do not directly relate to the start and end states of the event. In this manner, both the textual and video modalities have an event-oriented start, end, and change of state in the cross-modal semantic state space. Aligning them encourages cross-modal semantic consistency, enabling more precise temporal localization.

Specially, we extract semantic information from static frames at regular intervals using a pre-trained vision–language model. However, the semantic information from static frames only reflects the semantics of the current frame and does not capture changes in semantic states effectively. Recently, large language models have demonstrated powerful language understanding and generalization capabilities, enabling the generation of high-quality natural language responses. Therefore, we leverage pre-trained models such as CLIP and LLaMa2 for knowledge distillation, utilizing the broad, universal knowledge embedded within these models. This ensures our approach is adaptable and effective across a diverse range of contexts. We extract knowledge from a pre-trained large language model by inputting the semantic information from static frames in video features, allowing us to obtain high-quality descriptions of semantic changes and project them into a cross-modal semantic state space. For the predicted proposal *T* = {$t^{s}$, $t^{e}$}, we map the projected static features at times $t^{s}$ and $t^{e}$ to represent the start and end states, respectively. We consider the semantic changes from $t^{s}$ to $t^{e}$ as indicating an “event” and build a video-modal state set that maintains semantic coherence. Additionally, we extract potential event start and end states based on knowledge from the language model to establish a “start–event (query sentence)–end” state set while maintaining semantic coherence for the text modality. Subsequently, we align the state sets of the two modalities in a cross-modal semantic state space. Then, our model reconstructs the original query by using the video features in the proposal. The reconstructed word distribution is used to compute the Kullback–Leibler divergence (relative entropy) of the distribution of the states’ similarity as well as the cross-entropy of the true word distribution of the original query. Through this approach, we enhance the event-oriented cross-modal semantic consistency, avoiding the false high correlations caused by irrelevant frames and queries. Simultaneously, we ensure semantic coherence from the beginning to the occurrence and end of events in both video and text modalities, facilitating the model’s understanding of the essence of events.

In summary, our method addresses the challenge of spurious high correlations of similar irrelevant video frames for weakly supervised temporal language grounding (WSTLG). The key innovations and contributions of our approach include:

- We propose a novel Event-oriented State Alignment Network (ESAN) to improve cross-modal alignment in weakly supervised temporal language grounding. Unlike existing methods that often overlook the need for event-oriented semantic coherence, our ESAN constructs a semantic state space that captures the progression of events from start to end. This ensures more accurate representation and alignment of the events described in natural language queries with corresponding video segments.
- We devise a semantic state alignment module that aligns the event-oriented “start–event–end” state sets constructed from two modalities to facilitate better understanding of the essence of event occurrences. Our method leverages entropy-based knowledge distillation to enhance the alignment of “start–event–end” state sets across modalities. This novel application of information theory principles improves the discriminative power of the model, particularly for distinguishing relevant frames from similar but irrelevant ones, which is often overlooked in existing methods.
- With significantly enhanced semantic coherence and consistency, ESAN achieves substantial performance improvements on both the Charades-STA and ActivityNet-Captions datasets compared to recent, well-established methods. This highlights the potential of our insight in weakly supervised temporal language grounding.

These contributions address critical gaps in existing methods and provide a comprehensive solution to the challenges of weakly supervised temporal language grounding, making our approach both innovative and effective.

## 2. Related Work

### 2.1. Fully Supervised Temporal Language Grounding

Temporal language grounding refers to the process of aligning a video segment with a natural language query, which enables it to handle more complex and mixed events. Since these queries arise from natural language statements in open domains, establishing robust interactions between video and text modalities becomes crucial. In fully supervised settings, previous research can be categorized into three main types. The first category employs a two-stage paradigm, where a series of candidate moment proposals are generated and subsequently scored for ranking. Early methodologies such as TALL [12] and MCN [13] employ techniques like sliding windows along the temporal dimension to produce proposals of varying lengths. Following this, multi-modal fusion and alignment techniques are applied to identify the most suitable proposal [3,14,15]. Some methods extend this approach by introducing deeper visual–textual interactions. Another category known as one-stage methods designs end-to-end structures to directly output the matching moment for a given sentence. For instance, ExCL [16] utilizes a tied LSTM span predictor to output start and end frames following cross-modal interactions. 2DTAN [17] creates a two-dimensional temporal map to cover diverse video segments with different lengths. This approach proves beneficial for learning the relationships within temporal contexts. Lastly, reinforcement-learning-based methods [18] involve training an agent to make decisions regarding the movement of start and end points along the temporal sequence. However, all these methods necessitate temporal boundary annotations as supervision during training, which can be labor-intensive and prone to errors due to the subjectivity of different annotators.

### 2.2. Weakly Supervised Temporal Language Grounding

To address the reliance on temporal boundary annotations, researchers are exploring weakly supervised temporal language grounding, which relies solely on video-level descriptions. Many of these methods draw on the multi-instance learning (MIL) paradigm. TGA [5] introduces text-specific global features for videos to conduct MIL and effectively computes cross-modal similarity. Meanwhile, WSLLN [4] focuses on measuring segment–text consistency and simultaneously conducts segment selection. Recognizing the effectiveness of modeling temporal relationships, some weakly supervised methods [7,19] adopt 2DTAN. They treat the 2D feature map as a bag and consider candidate moments as instances within an MIL framework. Another kind of weakly supervised temporal language grounding methods lies in the reconstruction-based approaches. They reconstruct the original query and optimize using a cross-entropy-based reconstruction loss function, which significantly enhances the accuracy of query reconstructions. This leads to more reliable temporal grounding results. For instance, SCN [8] generates multiple proposals and selects the top-k candidates that excel at reconstructing masked words in queries. On the other hand, CNM [9] and CPL [10] not only utilize reconstruction mechanisms but also incorporate negative samples for contrastive learning. These methods fuse multimodal information to predict a Gaussian mask as a proposal and mine challenging negative samples within the same video.

However, there is challenging problem with previous methods: false high correlations. These methods primarily learn the relevance between query sentences and video frames. Some frames that are not within the correct moment may still show certain relevance to vocabularies in the query sentence. Due to the lack of temporal annotations, such pseudo-relevant frames are not penalized, leading to false correlations that can interfere with the accuracy of event localization. To solve this issue, we hypothesize that improving event-oriented semantic coherence and consistency will prevent the model from being misled by false correlations from individual frames.

### 2.3. Pre-Trained Large Language Model

Recently, advancements in pre-trained large language models [20,21] have revolutionized natural language understanding and generation. Similarly, multi-modal pre-trained models in vision–language tasks [22,23,24] have demonstrated impressive performance in image–text comprehension, leveraging self-supervision and vast text data to achieve strong generalization capabilities.

However, deploying these large models in downstream applications often entails significant computational overhead due to fine-tuning requirements. Additionally, current multimodal models predominantly focus on static image–text relationships, exhibiting limited proficiency in capturing dynamic temporal information in videos.

To utilize pre-trained vision–language models with limited computing resources, our proposed ESAN model addresses these limitations by a knowledge distillation technique, which benefits the dynamic semantic state alignment in capturing event-oriented transitions accurately, thereby improving weakly supervised temporal language grounding.

## 3. Approach

**Figure 2:**
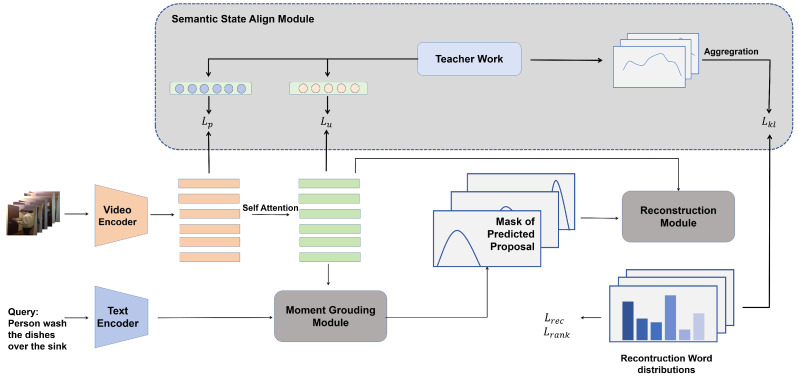
The overall architecture of our approach, including four components: feature encoder, moment grounding module, reconstruction module, and semantic state alignment module.

The objective of weakly temporal language grounding is to identify and retrieve the moment *T* = ($t^{s}$, $t^{e}$) within an untrimmed video that corresponds to a specified query. As shown in Figure 2, our reconstruction framework comprises four key components. Initially, the feature extractor is responsible for extracting visual and textual features from both the video and the query. These extracted features serve as inputs for the moment grounding module, which predicts the temporal proposal ($t^{s}$, $t^{e}$) of the corresponding events. Subsequently, the reconstruction module produces the reconstructed query by leveraging multi-modal information within the predict proposal to reconstruct the masked query. The video feature and reconstruction performance are supervised in a semantic state aligning module.

### 3.1. Feature Extraction

#### 3.1.1. Language Encoder

We extract the word embedding of a query $Q={\left\{q_{i}\right\}}_{i=0}^{L_{q}}$ using GloVe [25], and it is fed into a single-layer fully connected network. The word embedding $Q^{\prime }={\left\{q_{i}^{\prime }\right\}}_{i=0}^{N_{v}}$ interacts with visual feature in the following module, where d is the dimension of the feature and $L_{q}$ is the length of the sentence.

#### 3.1.2. Video Encoder

We extract the visual features from the given video using a pre-trained model, which helps us to capture spatial information effectively, even in the presence of noise. Then, fixed-interval sampling is performed on the vision features to divide $L_{v}$ frames of the video into $N_{v}$ clips. Each video clip consists of $\frac{L_{v}}{N_{v}}$ frames. Hence, the input video is denoted as a sequence of small video clips $V={\left\{v_{i}\right\}}_{i=0}^{N_{v}}\in R^{N_{v}*d}$, where the dimension of the visual feature d is same as that of the text feature.

### 3.2. Moment Grounding Module

Our method leverages transformer-based architectures and multi-head attention mechanisms for cross-modal fusion due to their superior adaptability and ability to establish long-range contextual relationships between contexts. We constructed a moment grounding module based on the standard transformer to predict the temporal segments in videos that match the queried events. The multi-head attention mechanism ensures that each video frame perceives contextual information from its surrounding frames, which helps to mitigate the impact of some noisy frames.

We first perform self-attention calculations on the features of video, highlighting the video tokens $V^{\prime }={\left\{v_{i}^{\prime }\right\}}_{i=0}^{N_{v}}$ that are considered to be context aware.

Assuming that we have the architecture of a transformer with an encoder Enc(·) and a transformer decoder Dec(·), we then perform cross-modal fusion using them, and we feed the fused features into a grounding head for predicting the moment of the event described in the query:

(1) $$\begin{array}{cc} F & =Dec(V^{\prime },Enc\left(Q^{\prime }\right))\\ c & =\sigma (W^{c}\cdot F)\\ l & =\sigma (W^{l}\cdot F) \end{array}$$

where $W^{c},W^{l}\in R^{d_{k}*1}$ are the learnable parameters, and σ means the sigmoid function. The prediction *T* =$\left(t^{s},t^{e}\right)$ of temporal grounding can be obtained as follow:

(2) $$\begin{array}{cc} t^{s} & =max\left(0,c-\frac{w}{2}\right)N_{v}\\ t^{e} & =min\left(1,c+\frac{w}{2}\right)N_{v} \end{array}$$

### 3.3. Reconstruction Module

Our method employs a reconstruction module to assist with temporal grounding. To select the best moment proposal in an end-to-end manner, we follow the reconstruction framework of CPL. Our method evaluates and selects proposals by reconstructing masked words in the query. Recognizing the importance of context, we believe that words containing key contextual information are closely related to video content. Consequently, only video segments that match the query can accurately reconstruct these key words. To enhance the reconstruction module’s capability, we randomly mask one-third of the words in the query and then use a mask conditional reconstructor to reconstruct the masked query $Q^{*}$, while we use cross-entropy and cross-entropy contrastive loss functions for model optimization. In the mask conditional reconstructor, the moment proposal is formulated as a mask that follows a Gaussian distribution:

(3) $$\begin{array}{c} m∼N(c,l^{2}) \end{array}$$

as our prediction. The mask *m* forces the attention in the transformers onto the context of the predicted proposal. Following CPL [10], we predict *K* proposals with mask $m_{i}$, where i={1,...,K}, and we select the one with the best reconstruction. Then, we reconstruct the masked query using the video feature $V^{\prime }$ and the mask *m*:

(4) $$\begin{array}{c} {\hat{Q}}_{i}=Softmax\left(W^{r}\cdot Dec(Q^{\prime },Enc\left(V^{\prime },m_{i}\right))\right) \end{array}$$

where ${\hat{Q}}_{i}$ denotes the reconstructed probability distribution of the *i*-th proposal, and $W^{r}$ is a trainable parameter. We also calculate the word distributions ${\hat{Q}}^{total},{\hat{Q}}_{i}^{neg}$ and use the masks representing the total video and the segments beyond the predicted proposal to reconstruct the masked query. Given the importance of accurate reconstruction for effective temporal grounding, we utilize a cross-entropy-based reconstruction loss function to optimize the module. This approach enhances the accuracy of the reconstructed queries, which is critical for selecting the optimal proposal in weakly supervised scenarios. Assuming the word distribution of the reconstruction result is $\hat{R}=\left\{p\left(r_{i}\right)\right\}$ and the real distribution of the original sentence is *Q*, we use the cross-entropy to calculate the distance between them:

(5) $$\begin{array}{c} L_{ce}\left(Q,\hat{R}\right)=-\sum \log\left(\hat{R}\left(q_{i}\right)\right),q_{i}\in Q \end{array}$$

We apply the reconstruction loss and ranking loss to enable our model to have comparative reconstructive capabilities.

(6) $$\begin{array}{cc} n & =\underset{1<=i<=K}{argmin}L_{ce}\left(Q,{\hat{Q}}_{i}\right)\\ L_{rec} & =L_{ce}\left(Q,{\hat{Q}}_{n}\right)+L_{ce}\left(Q,{\hat{Q}}_{total}\right)\\ L_{rank} & =max(L_{ce}\left(Q,{\hat{Q}}_{n}\right)-L_{ce}\left(Q,{\hat{Q}}^{total}\right)+\beta_{1},0)\\ \; & +max(L_{ce}\left(Q,{\hat{Q}}_{n}\right)-L_{ce}\left(Q,{\hat{Q}}_{k}^{neg}\right)+\beta_{2},0) \end{array}$$

where $\beta_{1},\beta_{2}$ are the hyperparameters to control the gaps between reconstruction performances.

### 3.4. Semantic State Alignment Module

In this module, we treat the large model as the teacher network and our grounding network as the student network for knowledge distillation, aiming to better leverage the generic knowledge in the large model to enhance the event-oriented semantic consistency of the grounding network.

#### 3.4.1. Principle Assumption

Our method is based on the assumption that only video segments whose states all align with the query are considered to be matched. We define the semantic states in a video as follows: at time t1, the video’s semantic state is termed as “$start_{v}$”, the aggregated semantic state from t1 to t2 is termed as “$event_{v}$”, and the semantic state at time t2 is termed as “$end_{v}$”. This forms a unimodal semantic state set for the video: $set_{v}=\left(start_{v},event_{v},end_{v}\right)$. For the query event $event_{q}$, we generate a corresponding unimodal semantic state set $set_{q}=\left(start_{q},event_{q},end_{q}\right)$ using knowledge distillation. Given the general knowledge within large models, $set_{q}$ maintains semantic coherence and temporal consistency.

If the video segment within (t1, t2) matches the query, each state in $set_{v}$ will correspond to a state in $set_{q}$. For non-matching segments, partial matches may occur. Our semantic state alignment module addresses the false high correlations caused by unrelated segments with similar semantic elements by ensuring that unrelated segments’ states $start_{v}$ and $start_{q}$ as well as $end_{v}$ and $end_{q}$ do not align.

#### 3.4.2. Event-Oriented Semantic State Space

**Figure 3:**
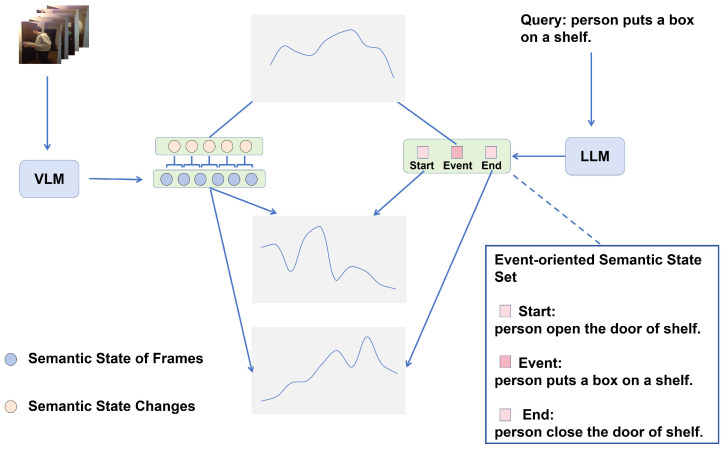
The generation of soft labels for the teacher network in the semantic state alignment module. We use a pre-trained vision–language model and a large language model to produce the event-oriented semantic state sets of two modalities. Then, we calculate the element-wise similarities between the sets we produced. The similar values will be used for supervising the reconstruction performance. By the way, the embedding of semantic states in the video modality will also be the target of alignment.

We construct an event-oriented semantic state space to implement semantic alignment, which is shown in Figure 3. Our goal is for the features from both the video modality and the text modality to be event-oriented, ensuring intra-modal semantic coherence and cross-modal semantic consistency. The former entails that feature projections in the semantic state space from both modalities adhere to the state transformation of “start–event–end”. The latter necessitates aligning state transformations from both modalities within the same semantic state space.

Pre-trained large language models trained on massive datasets possess strong language understanding and generation capabilities. We utilize pre-trained models that are trained on extensive and diverse datasets covering a wide range of general-purpose scenarios. By leveraging the universal knowledge from these models, we ensure that our method maintains high applicability and robustness across different scenes and domains.

However, directly fine-tuning large models requires high computational resources. Recognizing the resource intensity of training and fine-tuning large models, we utilize a knowledge extraction strategy to leverage the general knowledge embedded within these models. This approach enhances the accuracy of our smaller network without incurring significant resource costs.

Firstly, we construct prompting statements based on the event *Q* in the text modality, and we input them into the large language model to generate embeddings of start state $Q_{s}$ and end state $Q_{e}$ for the event. Considering that $Q_{s}$ and $Q_{e}$ are generated by universal prior knowledge in large models, we believe that $S^{q}=\left\{Q_{s},Q,Q_{e}\right\}$ satisfies semantic consistency about the event *Q*. This set denotes $set_{q}$ in the principle assumption.

For the video modality, we similarly obtain a soft description $O=\{o_{i}\}$ of semantic states by captioning the video at equidistant intervals using a visual–language model. This soft description only contains static information of individual frames and lacks the ability to understand dynamic changes. To capture the semantic state changes in the video, we construct prompting statements based on two adjacent soft descriptions $\left\{O_{i},O_{i+1}\right\}$, and we use a large language model to explicitly model the changes in the semantic states $c_{i}$ that occur during this time interval in the video. This process allows us to derive a set of semantic states $C=\{c_{i}\}$ for the video. Since *C* is generated based on continuous video soft descriptions, it certainly satisfies semantic coherence.

#### 3.4.3. Knowledge Distillation

In this part, the outputs of the teacher network serve as soft labels to calculate the relative entropy for our grounding network. Firstly, we project the video features from the student network into the cross-modal semantic state space and align their embeddings with the semantic state embeddings of the videos. This is carried out to reduce the semantic gap between the video features and the semantic state sets we have constructed:

(7) $$\begin{array}{cc} L_{p} & =|o_{i}-v_{i}|,0\leq i\leq N_{v}\\ L_{u} & =|c_{k}-v_{k}^{\prime }|,t^{s}<k<t^{e}\\ L_{align} & =L_{p}+L_{u} \end{array}$$

Note that the alignment loss in Equation (7) can be divided into two parts: $L_{p}$ and $L_{u}$, representing the separate alignments of image features with single-frame information and context-aware video features with soft descriptions of semantic state changes, achieving cross-modal semantic alignment from both static and dynamic perspectives.

Each proposal is predicted by the moment grounding module as pseudo-labels, and the set of semantic state oriented events in the video modality can be formulated as

(8) $$\begin{array}{c} S_{i}^{v}=\left\{o_{t_{k}^{s}},\left\{c_{i}\right\},o_{t_{k}^{e}}\right\},t_{k}^{s}<i<t_{k}^{e},1\leq k\leq K \end{array}$$

The set $S_{i}^{v}$ means the $set_{v}$ in the principle assumption. The semantic state sets of the same event *Q* in different modalities, $S^{q}$ and $S^{v}$, demonstrate semantic consistency. We compute the element-wise similarity between them, using it as soft labels from the teacher network to train the student network:

(9) $$\begin{array}{cc} {\hat{S}}_{k}^{s} & =\frac{Q_{s}\cdot o_{t_{k}^{s}}}{∥Q_{s}∥\cdot ∥o_{t_{k}^{s}}∥}\\ {\hat{S}}_{k}^{q} & =\underset{t_{k}^{s}<i<t_{k}^{e}}{max}\left(\frac{Q\cdot c_{i}}{∥Q∥\cdot ∥c_{i}∥}\right)\\ {\hat{S}}_{k}^{e} & =\frac{Q_{e}\cdot o_{t_{k}^{e}}}{∥Q_{e}∥\cdot ∥o_{t_{k}^{e}}∥}\\ {\hat{S}}_{k} & =({\hat{S}}_{k}^{s}+{\hat{S}}_{k}^{q}+{\hat{S}}_{k}^{e})/3,1\leq k\leq K \end{array}$$

For the *k*-th predicted proposal $\left\{t_{k}^{s},t_{k}^{e}\right\}$, ${\hat{S}}_{k}^{s},{\hat{S}}_{k}^{q}$, and ${\hat{S}}_{k}^{e}$ respectively represent the similarity of the static information at time $t_{k}^{s}$ to the start state of the event, the semantic state changes from $t_{k}^{s}$ to $t_{e}^{s}$ within the event, and the static information at time $t_{k}^{s}$ at the end state of the event. The reconstruction word probability ${\hat{Q}}_{k}$ of the student network’s output is used as an indicator for selecting the best proposal. We supervise the reconstruction word probability distribution of the student network’s output using ${\hat{S}}_{k}$ as soft labels to calculate relations, promoting semantic coherence and consistency between the videos covered by the proposals and the events queried. The distillation loss can be calculated by:

(10) $$\begin{array}{cc} {\bar{Q}}_{k} & =\sum p\left(q_{i}\right),q_{i}\in Q\\ L_{kl} & =\sum_{1=<k=<K}{\bar{Q}}_{k}log\left({\bar{Q}}_{k}-{\hat{S}}_{k}\right) \end{array}$$

where p(w) denotes the probability of the word w in $\hat{Q}$. Finally, we use the time points with the highest similarities between the start and end states in both modalities as pseudo-labels to guide the temporal localization of the best proposal with the smallest reconstruction loss:

(11) $$\begin{array}{cc} {\hat{y}}_{s} & =\underset{k}{argmax}{\hat{S}}_{k}^{s},0\leq k\leq N_{v}\\ {\hat{y}}_{e} & =\underset{k}{argmax}{\hat{S}}_{k}^{e},0\leq k\leq N_{v}\\ L_{loc} & =|{\hat{y}}_{s}-t^{s}|+|{\hat{y}}_{e}-t^{e}| \end{array}$$

### 3.5. Training and Inference

During the training phase, we update the parameters of the student network using the aforementioned losses:

(12) $$\begin{array}{c} L=L_{rec}+L_{rank}+L_{loc}+\alpha_{1}L_{align}+\alpha_{2}L_{kl} \end{array}$$

where $\alpha_{1}$ and $\alpha_{2}$ are the hyperparameters to balance the weights of different losses.

During the inference phase, among the *K* proposals generated by the moment grounding module, we select the proposal with the smallest $L_{rec}$ as the predicted event-matching moment.

## 4. Experiments

### 4.1. Datasets

To evaluate our method, we conducted experiments on temporal language grounding using two benchmark datasets.

**Charades-STA** [12] is a dataset that focuses on indoor daily activities and contains 12,408 moment–sentence pairs in the training set and 3720 pairs in the testing set. The average video length, moment length, and query length in Charades-STA are 30.60 s, 8.09 s, and 7.22 words, respectively. We report the results on the test set.

**ActivityNet-Captions** [26] is currently the largest dataset on TLG and consists of 20,000 untrimmed videos and over 70,000 pairs related to open-world activities. The average lengths of the target and untrimmed video moments is 37.1 and 117.6 s, respectively, with an average query length of 14.4 words. We present the results based on the $val_{2}$ split.

### 4.2. Metrics

All results are measured using the evaluation metric “R@1,IoU>*m*”, which indicates the percentage of output results for which the intersection over union (IoU) between the model’s best-matched query moment and the ground truth exceeds the threshold *m*. We separately used m={0.1,0.3,0.5} for ActivityNet-Captions and m={0.3,0.5,0.7} for Charades-STA.

### 4.3. Implementation Details

#### 4.3.1. Data Preprocessing

For the Charades-STA dataset, we apply I3D [27] features, while for the ActivityNet-Captions dataset, we use C3D [28] features. As for the natural language sentence queries, we utilize pre-trained GloVe [25] embeddings to extract 300-dimensional word embeddings.

#### 4.3.2. Experiment Settings

For knowledge distillation, we adopt frozen CLIP [29] and Llama2 [21] as the teacher network. The number of downsampled video clips for all datasets is set to 200. We utilize three attention layers with a 256-dimensional hidden state in the transformer model. During training, the batch size is set to 32 for all datasets, and we employ the Adam optimizer with an initial learning rate of 0.0004. We conduct the training and inference on only one NVIDIA RTX3090 GPU with 24 GB of memory.

### 4.4. Results and Comparisons

Table 1 and Table 2 present the results of our method alongside those of previous state-of-the-art methods on ActivityNet-Captions and Charades-STA.

**Comparison to the baseline**: We selected CPL [10] as the baseline to demonstrate the effectiveness of our method, given that we adopted the reconstruction framework of CPL.

In comparison, our method exhibits significant improvements on both datasets. Specifically, on Charades-STA, when considering accuracy at IoU = 0.3, 0.5, and 0.7, our approach achieves substantial improvements of 2.69%, 1.61%, and 1.59%, respectively.

**Comparison to SOTA methods**: As shown in Table 1, our model clearly achieves the highest performance across all metrics for Charades-STA. Additionally, for the Activitynet-Captions dataset, our method also demonstrates the best performance at IoU values of 0.3 and 0.5, with only a slight deviation below CPL at an IoU of 0.1. Our method consistently delivers strong performance on both datasets, indicating the potential of our approach.

Overall, the empirical results validate our assumption by demonstrating the effectiveness of our approach at both matching and distinguishing relevant and irrelevant video segments. This is consistent with previous research emphasizing the significance of semantic coherence and consistency in video analysis.

### 4.5. Statistical Testing

To demonstrate the robustness and reliability of our results, we conduct statistical tests and report the confidence intervals. We conduct rigorous statistical tests on three evaluation metrics across two datasets: Charades-STA and ActivityNet-Captions. The *p*-values obtained from these tests were all less than 0.05, indicating that the results are statistically significant. Moreover, we conduct multiple experiments and attain the 95% confidence intervals (CIs) for different IoU thresholds. The 95% confidence intervals are [67.53, 68.92] (IoU > 0.3), [49.95, 51.01] (IoU > 0.5), and [23.56, 24.35] (IoU > 0.7) for Charades-STA, while they are [78.33, 81.83] (IoU > 0.1), [55.37, 57.21] (IoU > 0.3), and [32.20, 33.51] (IoU > 0.5) for Activitinet-Captions. These confidence intervals demonstrate the robustness of our method across different IoU thresholds and datasets.

### 4.6. Ablation Study

To analyze the effectiveness of our method, we conduct an ablation study on the Charades-STA dataset.

**Effectiveness of knowledge distillation in semantic state alignment module**: We constructed ablation models by only conducting embedding alignment (Emb Align), state alignment (State Align), localization guiding (Loc Guide), and doing nothing (w/o Any). As shown in Table 3, the full model outperforms the ablation models in all the metrics. This can be attributed to our integration of the general knowledge of pre-trained large models, prompting the model to gain a deeper understanding of the semantic states changes of the described events. This improves the cross-modal semantic consistency. It is worth noting that the singular state alignment does not exhibit clear performance due to the lack of embedding alignment. There exists a semantic gap between the video features of the student network and the state sets in the semantic space, which leads to a lack of semantic coherence in the constructed state sets for event-oriented semantics, thereby hindering cross-modal semantic alignment.

**Effectiveness of event-oriented semantic state space**: Further, we evaluate the different ways to use the knowledge in large models to highlight the efficacy of our proposed event-oriented semantic state space. The ablation approach involves directly calculating the similarity between the queries and the captions of frames in the video without constructing an event-oriented semantic state space and proposing “start–event–end” sets of semantic states. As the results show in Table 4, our approach demonstrates significant advantages. This is because the captions of frame images only contain static visual information, which can easily produce false high correlations with some words in the query. In contrast, our method constructs a semantic state set of “start–event–end” for the query event, capturing coherent dynamic changes between semantic states. Cross-modal alignment based on these state changes improves cross-modal semantic consistency, which improves the accuracy of moment grounding.

### 4.7. Sensitivity Analysis

To analyze the sensitivity, we varied the weights of losses α1,α2 to observe the accuracy with IoU thresholds of 0.3, 0.5, and 0.7 while keeping the other hyperparameters unchanged.

**Figure 4:**
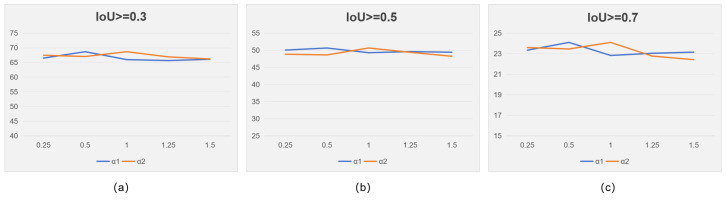
The results with different values of α1 and α2. Subfigures (**a**–**c**) shows the results when IoU>0.3,0.5 and 0.7.

As shown in Figure 4, our model exhibits stability and robustness across different hyperparameter values. Specifically, our model achieves optimal performance when α1=0.5 and α2=1; exceeding these values leads to a slight decline in performance. This is attributed to the excessive reliance on soft labels when α1,α2 are too large, neglecting the importance of reconstruction performance for selecting the best proposals.

### 4.8. Discussion about Complexity

**Comparison with large pre-trained models:** The common knowledge within large pre-trained language models benefits our semantic state alignment. However, fine-tuning large models is resource-intensity. Thus, we efficiently leverage the general knowledge within these models by employing a knowledge extraction strategy, minimizing resource consumption. Our experiments indicate that this process requires limited resources and can be performed on a single NVIDIA RTX 3090 GPU, highlighting the practicality and scalability of our approach.

In constructing the cross-modal semantic state space for events, we employ LLaMa2-7B [21] as the teacher network. Thus, we compare its complexity against our own network. Since LLaMa2-7B, being a language model, does not possess visual perception capabilities, we input the semantic embeddings of video frames in chronological order to facilitate temporal grounding. Experiments are carried out on the Charades-STA dataset using an NVIDIA RTX 3090, and the results are presented in Table 5. The outcomes clearly demonstrate that our model has only 1/57 of the parameter size of Llama2-7B, resulting in a significantly faster inference speed by 268 times. This suggests that our model can effectively harness the general knowledge of large models through knowledge distillation, making it more suitable for scenarios with limited computational resources.

**Space and time costs:** Table 6 demonstrates that by utilizing transformer-based architectures and multi-head attention mechanisms, our model exhibits relatively low time and space overhead during both training and inference. Specifically, with a batch size of 32, the memory consumption during training the small model is approximately 6.7 GB, knowledge distillation requires about 13 GB, and inference uses about 3.2 GB. Additionally, on a single NVIDIA RTX 3090 GPU, the inference time per sample is around 0.25 s. These results highlight the efficiency of our model, making it feasible for near-real-time applications even in environments with limited computational resources.

### 4.9. Qualitative Results

**Figure 5:**
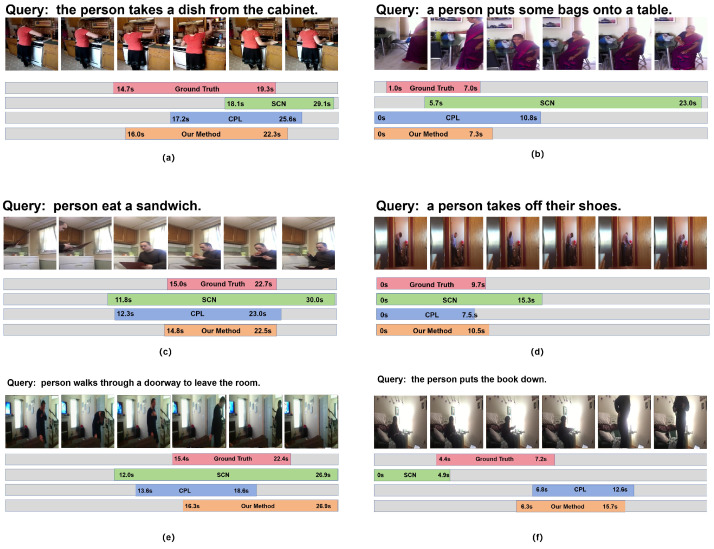
The qualitative results on Charades-STA. Subfigures (**a**–**d**) show the successful example of our model. Subfigures (**e**,**f**) show the cases of failure.

Figure 5 shows some qualitative results of our approach. Figure 5a,b demonstrates that our method is capable of better cognition and understanding of video content and the events described in the natural language queries, thereby enabling more precise localization. This is attributed to our model’s semantic coherence and consistency at avoiding being misled by the spurious high similarities of some unrelated frames. Additionally, as shown in Figure 5c,d, our method demonstrates more precise boundaries, which is attributed to the construction of the “start–event–end” semantic state set, which facilitates the identification of the temporal boundaries associated with the start and end states, thereby enhancing moment grounding accuracy.

Figure 5e shows that the model achieves rough localization but lacks precision. The query “person walks through a doorway to leave the room” is misinterpreted by the model, which inaccurately associates the ending state “leave the room” with the overall event state, leading to deviations. Another example, Figure 5f, showcases a clear misalignment due to the essential semantic element “book” not being prominent in the video frames. This absence results in incorrect cross-modal alignment and highlights a failure case.

These examples underscore both the strengths and limitations of our model, providing valuable insights into its performance across different scenarios.

## 5. Future Works

In this section, we outline several areas of future research that address the challenges posed by multi-language queries.

**Addressing multi-language queries**: As current datasets and prior work predominantly focus on English, expanding to multi-language queries presents unique challenges. Different languages exhibit distinct grammar, vocabulary, and expression styles, leading to potential information loss or misinterpretation. This also brings mixed-code [39] and code-switching [40], adding an additional layer of complexity. Moreover, supporting multiple languages often requires different models or adaptations, posing challenges for the scalability and generalization of the system.

To tackle these issues, future work will explore the use of language identification techniques and multilingual pre-trained models as text pre-processing. This approach aims to enhance the model’s adaptability and scalability, ensuring effective performance in diverse linguistic contexts.

**Addressing insignificant but important video elements**: Addressing the issue of reduced accuracy when semantic elements are not prominently featured in video frames is another focus. Enhancing the model’s robustness in such scenarios could involve integrating additional visual context or employing more sophisticated attention mechanisms to better capture subtle semantic cues.

## 6. Conclusions

In conclusion, this paper introduces a novel Event-oriented State Alignment Network (ESAN) that makes several unique contributions to the field of weakly supervised temporal language grounding. Here are the novel and unique contributions of ESAN:

Firstly, ESAN constructs event-oriented “start–event–end” semantic state sets for both textual and video data. This approach ensures that the model captures the complete sequence of state changes, thereby aligning text more accurately with the true essence of queried events.

Secondly, by applying relative entropy for knowledge distillation from pre-trained large models, ESAN achieves effective cross-modal alignment with various domains. This innovative use of information theory principles allows the model to leverage the general knowledge in pre-trained models while maintaining semantic coherence and consistency across modalities.

Then, ESAN’s methodology enables a deeper understanding of queried events based on semantic state changes rather than relying solely on superficial correlations between words and video frames. This leads to better intra-modal semantic coherence and cross-modal semantic consistency during the state transition process.

Experiments conducted on two widely-used datasets demonstrate that ESAN significantly outperforms existing methods in weakly supervised TLG. The results highlight the robustness and effectiveness of our proposed approach at capturing event-oriented semantics and reducing false positives caused by irrelevant frames.

However, our approach also has some limitations that must be considered. One notable challenge is handling multilingual queries. The current model may struggle with queries in different languages due to potential inconsistencies in semantic interpretation. Additionally, the accuracy of our method can diminish when essential semantic elements are not prominently featured in the video frames, leading to potential misalignments and decreased performance. Future work will focus on exploring methods to address these limitations.

## Figures and Tables

**Table 1:** Performance comparison on the Charades-STA dataset (IoU>m∈{0.3,0.5,0.7}). The best results are highlighted in **bold**, and the second-best results are underlined.

Method	IoU = 0.3	IoU = 0.5	IoU = 0.7
TGA [5]	32.14	19.94	8.84
CTF [30]	37.8	27.3	12.9
SCN [8]	42.96	23.58	9.97
DCCP [31]	-	29.8	11.9
WSTAN [7]	43.39	29.35	12.28
LoGAN [6]	48.04	31.74	13.71
MARN [32]	48.55	31.94	14.81
VCA [33]	58.58	38.13	19.57
CRM [34]	53.66	34.76	16.37
LCNet [35]	59.60	39.19	18.87
RTBPN [19]	60.04	32.36	13.24
CNM [9]	60.04	35.15	14.95
CPL [10]	65.99	49.05	22.61
Ours	68.68	50.66	24.10

**Table 2:** Performance comparison on ActivityNet-Captions dataset (IoU>m∈{0.1,0.3,0.5}). The best results are highlighted in **bold**, and the second-best results are underlined.

Method	IoU = 0.1	IoU = 0.3	IoU = 0.5
DCCP [31]	-	41.6	23.2
WS-DEC [36]	62.71	41.98	23.34
RGL [37]	65.99	44.49	24.33
VCA [33]	67.96	50.45	31.00
EC-SL [38]	68.48	44.29	24.26
MARN [32]	-	47.01	29.95
SCN [8]	71.48	47.23	29.22
RTBPN [19]	73.73	49.77	29.63
CTF [30]	74.2	44.30	23.6
WSLLN [4]	75.4	42.8	22.7
LCNet [35]	78.58	48.49	26.33
WSTAN [7]	79.78	52.45	30.01
CPL [10]	**82.55**	55.73	31.37
CRM [34]	81.61	55.26	32.19
CNM [9]	78.13	55.68	33.33
Ours	80.21	56.76	33.41

**Table 3:** The effectiveness of knowledge distillation in the semantic state alignment module for moment localization on the Charades-STA dataset. The **bold** means the best score.

Method	IoU = 0.3	IoU = 0.5	IoU = 0.7
Full Model	68.68	50.66	24.10
Emb Align	66.62	49.24	23.50
State Align	65.71	49.30	23.02
Loc Guide	66.66	49.62	24.12
w/o Any	65.62	49.21	22.20

**Table 4:** The importance of the event-oriented semantic state set (ESS) on the Charades dataset.The **bold** means the best score.

Method	IoU = 0.3	IoU = 0.5	IoU = 0.7
Full Model	68.68	50.66	24.10
w/o ESS	63.12	48.36	21.13

**Table 5:** Complexity comparison with pre-trained large model.

Method	Parameter Size	Time Cost
Our Model	13 M	0.25 s
LLAMA2-7B	745 M	6.7 s

**Table 6:** Space and time costs of our model on Charades-STA.

Method	Memory Usage	Time Cost
Training	6773 MB	1.12 s
Inference	3223 MB	0.25 s

## Data Availability

The data presented in this study are openly available in an open-access repository at [9]. Only publicly available data sources have been used, and thus no ethical permission is required.

## Abbreviations

The following abbreviations are used in this manuscript:

TLG	Temporal Language Grounding
ESAN	Event-oriented State Alignment Network
TALL	Temporal Activity Localization via Language
MCN	Moment Context Network
ExCL	Extractive Clip Localization
LSTM	Long Short-Term Memory
2DTAN	2-dimensional Temporal Adjacent Networks
MIL	Multi-Instance Learning
TGA	Text-specific Global features Alignment
WSLLN	Weakly Supervised Language Localization Network
SCN	Semantic Complement Network
CNM	Contrastive Negative sample Mining
CPL	Contrastive Proposal Learning
IoU	Intersection over Union
CLIP	Contrastive Language-Image Pre-training
